# Can individual cognitions, self-regulation and environmental variables explain educational differences in vegetable consumption?: a cross-sectional study among Dutch adults

**DOI:** 10.1186/s12966-014-0149-1

**Published:** 2014-12-06

**Authors:** Linda Springvloet, Lilian Lechner, Anke Oenema

**Affiliations:** Department of Health Promotion, School for Public Health and Primary Care (CAPHRI), Maastricht University, P.O. Box 616, 6200 MD Maastricht, The Netherlands; Faculty of Psychology and Educational Sciences, Open University of the Netherlands, P.O. Box 2960, 6401 DL Heerlen, The Netherlands

**Keywords:** Vegetable consumption, Socio-economic status, Individual cognitions, Self-regulation, Physical environmental factors

## Abstract

**Background:**

Educational differences in health-related behaviors, where low- and moderate-educated individuals have poorer outcomes than high-educated individuals, are persistent. The reasons for these differences remain poorly understood. This study explored whether individual cognitions, self-regulation and environmental-level factors may explain educational differences in vegetable consumption.

**Methods:**

A cross-sectional study was conducted among 1,342 Dutch adults, of whom 54.5% were low/moderate-educated. Individuals completed an online questionnaire, assessing education, vegetable consumption, demographics, individual cognitions (attitude towards consuming 200 grams of vegetables a day, self-efficacy, subjective norm, intention, perception of vegetables as being expensive), self-regulation (general self-regulation, vegetable-specific action- and coping planning) and environmental-level factors (perception of availability of vegetables in the supermarket and availability of vegetables at home). The joint-significance test was used to determine significant mediation effects.

**Results:**

Low/moderate-educated individuals consumed less vegetables (M = 151.2) than high-educated individuals (M = 168.1, β = −0.15, *P* < .001). Attitude and availability of vegetables at home were found to partially mediate the association between education and vegetable consumption (percentage mediated effect: 24.46%).

**Discussion:**

Since attitude and availability of vegetables at home partially explain the difference in vegetable consumption between low/moderate- and high-educated individuals, these variables may be good target points for interventions to promote vegetable consumption among low/moderate-educated individuals.

## Background

Consuming a sufficient amount of vegetables can decrease the risk of multiple diseases, like cardiovascular diseases (CVD) and some forms of cancer [1-3]. In most Western countries, like The Netherlands, UK and USA, vegetable consumption is, however, far below recommended consumption levels [1,2,4,5]. Dutch adults, for example, have an average daily vegetable consumption of only 130 grams, while it is recommended to consume at least 200 grams of vegetables a day [2].

Vegetable consumption is even lower among lower educated individuals compared to high-educated individuals [2,6-9]. In the Netherlands, the difference in vegetable consumption is most striking between low/moderate- and high-educated adults; high-educated adults consume on average 147 grams of vegetables a day, while low- and moderate-educated adults consume on average only 122 and 127 grams respectively [2]. To be able to decrease the difference in vegetable consumption between low/moderate- and high-educated individuals, it is important to understand which factors may explain this difference.

Potential explanatory factors may be derived from motivational (e.g. Theory of Planned Behavior (TPB) [10], Social Cognitive Theory [11]), volitional (e.g. Self-Regulation Theory [12]) and socio-ecological models (e.g. the EnRG framework [13]). These models are mostly used to explain health-related behavior, but educational differences in factors derived from these models may also (partially) explain differences in vegetable consumption between low/moderate- and high-educated individuals.

According to the TPB [10], attitude, subjective norm and perceived behavioral control (or self-efficacy) predict intention, which subsequently predicts behavior (e.g. vegetable consumption) [14]. There is compelling evidence that attitude and self-efficacy explain vegetable consumption (e.g. [15,16]). There is also evidence that demonstrates educational differences in general self-efficacy [17] and perceived barriers related to vegetable consumption [18]. However, to our knowledge, there are no published studies that have studied the TPB variables as potential mediators in the association between vegetable consumption and education in adults. A study that addressed these variables for fruit consumption among adolescents, however, showed that perceived importance of healthy behaviors and self-efficacy mediated the relationship between fruit consumption and maternal education [19].

Self-regulation (the capacity to regulate and adapt behavior in order to achieve self-set goals) is also a potentially important determinant of behavior [12] that most likely has its effect in the post-motivational phase of the behavior change process. A study of Anderson et al. [20] found that enactment of self-regulatory behaviors, like ‘regulating vegetable consumption’, increased vegetable consumption. More specific self-regulation skills like action- and coping planning have also been found to be associated with vegetable consumption [21]. To our knowledge, there are no studies that have reported on educational differences in these skills for vegetable consumption or general dietary behaviors, but for smoking cessation it has been found that high-educated individuals are more likely to form instrumental and thus better quality coping plans than lower educated individuals [22]. Such educational differences in self-regulation may also exist in relation to vegetable consumption and could potentially explain educational differences in vegetable consumption.

With respect to environmental-level factors, physical environmental factors such as perceived costs of vegetables [9,23], perceived local availability of vegetables [9] and availability of vegetables at home [24,25] have been found to be associated with vegetable consumption. In addition, these factors have been suggested to explain the relation between education and vegetable consumption. A study of Inglis and colleagues [9] showed that perceptions of food availability, accessibility and affordability almost fully explain educational differences in vegetable consumption. Another study also showed that the relative importance of price in food choices (partially) mediated the effect of education on vegetable consumption among women [26]. A second category of potentially important physical environmental factors is availability of vegetables at home. A study among adolescents, for example, has shown that (perceived) accessibility of vegetables at home mediates the association between adolescents’ vegetable consumption and their parents’ education [25].

The aim of the present study is to examine whether individual cognitions (attitude, self-efficacy, subjective norm, intention, perception of vegetables as being expensive), self-regulation (general self-regulation and vegetable-specific action- and coping planning) and physical environmental factors (availability of vegetables at home and perception of availability of vegetables in supermarkets) mediate the relation between education and vegetable consumption in Dutch adults.

## Methods

### Study design and participants

A cross-sectional study was conducted, using baseline data from a randomized controlled trial aimed at testing the efficacy of a web-based, computer-tailored nutrition education intervention [27]. The Medical Ethics Committee of the Erasmus Medical Centre in Rotterdam, the Netherlands, approved the study protocol of the trial (NL35430.078.11/MEC-2010-408).

The target group of the study consisted of low/moderate- and high-educated Dutch adults (20–64 years). Individuals were recruited between March and October 2012 in five cities in the South of the Netherlands (Heerlen, Roermond, Venlo, Venray and Weert), mainly via personal mailings that were sent to 26,402 random home-addresses. Additionally, Facebook advertisements, advertisements in (local) newspapers, local television and distributing flyers and talking to people in shopping malls were used for recruitment. Individuals could sign up for the study by phone, e-mail or via the study website. Inclusion criteria were: aged between 20 and 65 years, having a sufficient understanding of the Dutch language (in reading and writing) and having Internet access. Exclusion criteria were: being on a diet prescribed by a physician or dietician, having a medical condition that implies restrictions in eating behavior (e.g. CVD or bowel disease) and not willing to sign an informed consent.

**Figure 1:**
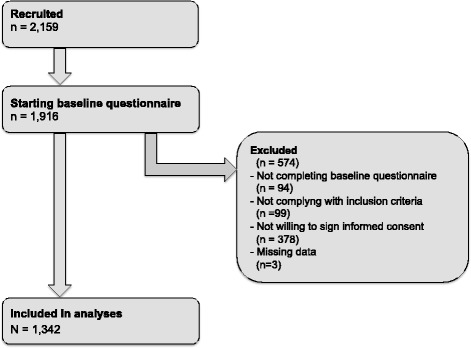
**Flow-chart of inclusion of respondents.**

A total of 2,159 individuals indicated to be willing to participate in the trial, of whom 1,345 were included in the study (Figure 1). Three individuals were excluded from the analyses, because of missing data. This leaves a total of 1,342 individuals to be included in the analyses (=62.2% of the registries).

### Procedure

Individuals who signed up for the study were sent an online baseline questionnaire [27]. One e-mail reminder for filling out the baseline questionnaire was sent two weeks after the initial invitation. The baseline questionnaire started with assessing the inclusion- and exclusion criteria. Individuals who met the inclusion criteria were asked to give online informed consent before they could continue with the baseline questionnaire. A written informed consent form was sent via postal- or e-mail and only individuals who signed and returned the written informed consent form were included in the study. For this study we only used the questions that assessed vegetable consumption, education, some background variables, like age and ethnicity, and potential mediators related to vegetable consumption (individual cognitions, self-regulation concepts, environmental-level factors). The questionnaire also included assessments of (determinants of) fruit, high-energy snack and fat intake. These dietary behaviors were not examined in this study, because education was found to predict vegetable consumption only.

### Measures

#### Vegetable consumption

Vegetable consumption was measured with a validated food frequency questionnaire consisting of four items using a reference period of one month [28,29]. Individuals were asked on how many days per week they usually consume cooked and raw vegetables or salads (ranging from 0–7 days per week) and how many tablespoons of cooked and raw vegetables or salads they usually ate on these days (ranging from one to six or more). Grams of vegetables per day were calculated by multiplying the frequency by the amount of tablespoons, divided by seven (days a week).

#### Education

To assess education, individuals had to indicate their highest attained education [30]. Answer possibilities were: 1) no education (primary school not finished); 2) primary school; 3) lower or preliminary vocational education; 4) lower general secondary education; 5) intermediate vocational education; 6) higher secondary or pre-university education; 7) higher vocational education; 8) university. Education was dichotomized into two educational groups; (0) high-educated (higher vocational education and university) and (1) low/moderate-educated (no education to higher secondary or pre-university education).

#### Potential mediators

Attitude towards consuming 200 grams of vegetables a day was measured with three items that assessed beliefs about health, taste and importance (Cronbach’s alpha (α) = 0.75) (Table 1). The item perception of vegetables as being expensive did not fit into the attitude scale and was therefore included in the analyses as a separate attitude item. Self-efficacy expectations were measured with two items about perceived difficulty and ability of consuming 200 grams of vegetables a day (Pearson correlation (r) = 0.63, *P* < .001). Social influence was assessed with one item relating to subjective norm and one relating to perceived vegetable consumption by others (modelling) (r = 0.57, *P* < .001). Intention was assessed with one item, asking whether the individual intended to consume 200 grams of vegetable a day. All individual cognitions [10] were measured on a 5-point scale. When Cronbach’s alpha was larger than 0.70, or correlation was significant, items were collapsed to a single variable by calculating the mean score over the different items.

**Table 1:** **Assessment of potential mediators**

**Concept**	**Items**	**Answer categories**	**α**
*Individual cognitions*			
Attitude towards consuming 200 grams of vegetables a day^a^	I think eating 200 grams of vegetables per day is …	Very unhealthy (1) – very healthy (5)	0.75
		Very unimportant (1) – very important (5)	
		Very disgusting (1) – very delicious (5)	
Perception of vegetables as being expensive^a^	I think eating 200 grams of vegetables per day is …	Very expensive (1) – very cheap (5)	N.A.
Self-efficacy^a^	Do you think you can eat 200 grams of vegetables per day in the next 6 months if you really want to?	Definitely not (1) – definitely (5)	0.73
	How difficult or easy do you think it is to eat 200 grams of vegetables each day in the next 6 months?	Very difficult (1) – very easy (5)	
Subjective norm^a^	Most people who are important to me…	Definitely not (1) – definitely (5)	0.77
	… Think I should eat 200 grams of vegetables each day		
	… Eat 200 grams of vegetables each day		
Intention^a^	Do you intend to eat 200 grams of vegetables a day?	Definitely not (1) – definitely (5)	N.A.
*Self-regulation concepts*			
General self-regulation^a^	When I have a goal, I can usually plan how to reach it	Definitely not (1) – definitely (5)	0.79
	I give up quickly (reversed)		
	I have trouble to make plans that help me to reach my goals (reversed)		
	I have a hard time setting goals for myself (reversed)		
	I set goals for myself and keep track of my progress		
	If I try to change something, I pay attention on how I am doing		
Action planning^b^	I have a clear plan for…	Completely disagree (1) – completely agree (4)	0.91
	… When I am going to eat more vegetables		
	… Which kinds of vegetables I am going to eat more		
	… How much vegetables I am going to eat more		
Coping planning^b^	I have a clear plan for…	Completely disagree (1) – completely agree (4)	0.92
	… What I am going to do when something interferes with my plans to eat more vegetables		
	… What I am going to do in situations in which it can be more difficult to eat more vegetables		
*Environmental-level factors*			
Perception of availability in supermarket^a^	In the store where I usually do my shopping, there is a sufficient amount of vegetables available	Completely disagree (1) – completely agree (5)	N.A.
Availability of vegetables at home^a^	How often do you have vegetables available at home?	Never (1) – always (5)	N.A.

α = Cronbach’s alpha.

^a^Measured on a 5-point scale (ranging from 1 to 5); ^b^Measured on a 4-point scale (ranging from 1 to 4).

General self-regulation was measured using six items of the Self-Regulation Questionnaire with the highest factor loading [31]. These items (α = 0.79) measured different aspects about ease or difficulty of making goals and plans, monitoring and perseverance. Answers were on a 5-point scale, ranging from definitely not to definitely. Action planning was measured with three items adapted from a questionnaire used in a study of Sniehotta et al. [32]. These items measured whether individuals have clear plans for when, how much and which vegetables to eat more (α = 0.91). Coping planning [32] was measured by assessing whether individuals have clear plans for what to do in difficult situations and when something interferes with their plans (r = 0.84, *P* < .001). Action- and coping planning were measured on a 4-point scale, ranging from completely disagree to completely agree. When Cronbach’s alpha was larger than 0.70, or correlation was significant, items were collapsed to a single variable by calculating the mean score over the different items.

The perception of the availability of vegetables in the supermarket where someone usually does the shopping was measured by assessing whether individuals perceive the availability of vegetables in their supermarket as sufficient. The availability of vegetables at home was assessed by questioning how often individuals have vegetables available at home. The environmental-level factors were measured on a 5-point scale.

#### Confounding variables

Potential confounding variables that were taken into account in this study are: age (in years), sex (male vs. female), place of residence (‘What is your place of residence?’: Heerlen, Roermond, Venlo, Venray, Weert) and ethnicity (non-Western vs. Western). Ethnicity was measured by asking the country of birth of one’s father and mother (Netherlands, Suriname/Netherlands Antilles, Turkey, Morocco or different). In line with the procedures of Statistics Netherlands [33] an individual was considered to be of Western ethnicity if both parents were born in Europe (except for Turkey), North America, Oceania, Indonesia or Japan. If at least one parent was born elsewhere, the individual was considered to be of non-Western ethnicity. Hence, a dichotomous measure of ethnicity was constructed: non-Western (0) and Western (1).

### Data analyses

Descriptive statistics were used to describe the study sample and the low/moderate- and high-educated subgroups. Independent t-tests and chi-square tests were conducted to examine differences in vegetable consumption, potential mediators and potential confounding variables between low/moderate- and high-educated individuals.

**Figure 2:**
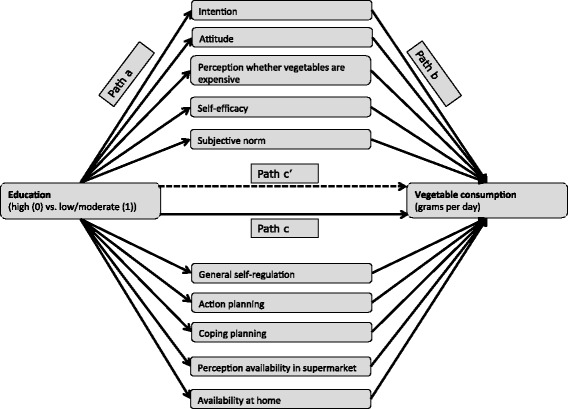
**Conceptual model for potential mediators between education and vegetable consumption.** Note: Path a = associations between education and potential mediators; path b = associations between potential mediators and vegetable consumption, adjusted for education; path c = total association between education and vegetable consumption; path c’ = direct association between education and vegetable consumption, adjusted for all potential mediators. All paths were adjusted for potential confounders (age, sex, place of residence, ethnicity).

For the mediation analysis, the model depicted in Figure 2 was used. Path c in this model refers to the association between education and vegetable consumption; path a refers to the associations between education and potential mediators; path b refers to associations between potential mediators and vegetable consumption, adjusted for education, because education may be a confounder in this association; path c’ refers to the association between education and vegetable consumption, adjusted for all potential mediators.

Mediation between education and vegetable consumption was established using the joint-significance test [34], which means that a variable is a mediator when both path a and path b are significant. The associations between education and potential mediators (path a) were assessed with multiple linear regression analyses. The associations between potential mediators and vegetable consumption, adjusted for education (path b) were assessed with one multiple linear regression analysis including all potential mediators in order to test these associations independent of the effects of other potential mediators.

The total effect of education on vegetable consumption (path c) was examined by a multiple linear regression analysis. The direct effect of education on vegetable consumption (path c’) was examined using one multiple linear regression analysis, adjusted for all potential mediators. Calculating path c and path c’ is not part of the joint-significance test, but differences between these pathways were used to determine whether there was full- or partial mediation [35]. Additionally, the percentage mediated effect was calculated by dividing the total mediated or indirect effect (sum of path a × path b for all significant mediators) by the total effect assessed in path c. For mediators that consist of multiple items, the percentage mediated effect of the separate items was also calculated.

The results of the linear regression analyses were verified using the bootstrapping method. With bootstrapping the study sample is resampled over and over again (n = 1,000), the regression coefficient for each sub-sample is calculated and a total standard error is provided, based on which a significance test is computed [36].

All regression analyses were adjusted for potential confounding variables (age, sex, place of residence and ethnicity). Alpha was set at .05. Analyses were performed with SPSS version 19 (IBM Corp, Armonk, NY, USA).

## Results

### Participant characteristics

A total of 1,342 individuals were included in the analyses. The mean age of the individuals was 49.02 years (SD = 10.63), 1.3% had a non-Western ethnicity and 35.5% were male (Table 2). 731 individuals were categorized as low/moderate-educated and 611 individuals as high-educated. The low/moderate-educated group had a higher mean age (*P* < .001) and consisted of fewer males (*P* = .01) than the high-educated group. The mean daily vegetable consumption was 158.9 grams (SD = 69.30). Low/moderate-educated individuals had a lower daily vegetable consumption (M = 151.2, SD = 69.02) than high-educated individuals (M = 168.1, SD = 68.58, *P* < .001). Overall, individuals had a positive, high, score on individual cognitions, except for the perception of vegetables as being expensive, which was scored as neutral (not expensive / not cheap). Positive, high, scores were also found for general self-regulation, perception of availability of vegetables in the supermarket and availability of vegetables at home. Low/moderate-educated individuals had a slightly less positive attitude towards consuming 200 grams of vegetables a day (*P* = .02*)*, a slightly lower score on general self-regulation (*P* < .001*)* and reported to have vegetables available at home slightly less often (*P* = .007*)* than high-educated individuals. Overall, individuals reported to not often have action- or coping plans, but low/moderate-educated individuals more often had action- (*P* < .001) and coping plans (*P* = .03*)* for eating more vegetables compared to high-educated individuals.

**Table 2:** **Descriptives and educational differences for demographics, vegetable consumption and potential mediators**

		**Total group (n = 1,342)**	**High-educated (n = 611)**	**Low/moderate-educated (n = 731)**	**T**	**X** ^**2**^	**Df**	***P***
Age in years (SD)		49.02 (10.63)	47.23 (11.34)	50.52 (9.76)	−5.63	-	1,211.05	<.001*
Sex (% male)		35.5	39.1	32.4	-	6.52	1	.01*
Place of Residence (%)					-	9.09	4	.06
	Heerlen	24.0	23.4	24.5	-	-	-	-
	Roermond	16.0	18.3	14.1	-	-	-	-
	Venlo	22.5	24.2	21.1	-	-	-	-
	Venray	18.9	17.5	20.0	-	-	-	-
	Weert	18.6	16.5	20.4	-	-	-	-
Ethnicity (% non-Western)		1.3	0.8	1.8	-	2.32	1	.13
Vegetable consumption		158.9 (69.30)	168.1 (68.58)	151.2 (69.02)	4.46	-	1,340	<.001*
Individual cognitions								
	Attitude^a^	4.16 (0.49)	4.20 (0.48)	4.13 (0.50)	2.44	-	1,340	.02*
	Perception of vegetables as being expensive^a^	3.01 (0.51)	3.02 (0.53)	3.00 (0.50)	0.95	-	1,340	.34
	Subjective norm^a^	3.45 (0.82)	3.45 (0.83)	3.45 (0.81)	−0.03	-	1,340	.97
	Self-efficacy^a^	3.84 (0.84)	3.82 (0.84)	3.84 (0.83)	−0.46	-	1,340	.65
	Intention^a^	4.06 (0.89)	4.08 (0.90)	4.04 (0.89)	0.77	-	1,340	.44
Self-regulation concepts								
	General self-regulation^a^	3.48 (0.78)	3.58 (0.77)	3.39 (0.79)	4.65	-	1,340	<.001*
	Action planning^b^	2.24 (0.64)	2.16 (0.65)	2.30 (0.62)	−3.91	-	1,340	<.001*
	Coping planning^b^	2.10 (0.64)	2.06 (0.66)	2.13 (0.63)	−2.15	-	1,340	.03*
Environmental-level factors								
	Perception of availability in supermarket^a^	4.47 (0.65)	4.46 (0.64)	4.48 (0.66)	−0.37	-	1,340	.71
	Availability of vegetables at home^a^	4.54 (0.61)	4.59 (0.56)	4.50 (0.64)	2.72	-	1,336.11	.007*

T = Independent t-test; X^2^ = Chi-square test; Df = Degrees of freedom; *Significant difference between educational levels (*P* < .05).

^a^Answer scale ranging from 1 to 5; ^b^Answer scale ranging from 1 to 4.

### Joint-significance test

The results of the associations in path a and path b (the joint-significance test) are shown in Table 3. Even though there were a number of significant associations between education and potential mediators and between potential mediators and vegetable consumption, only attitude and availability of vegetables at home could be identified as mediators in the association between education and vegetable consumption, since for these two variables both path a and path b were significant.

**Table 3:** **Associations between education and potential mediators (path a)** ^**a**^ **and potential mediators and vegetable consumption (path b)** ^**a,b**^

**Potential mediators**		**Standardized regression coefficient path a** ^**a**^		**Standardized regression coefficient path b** ^**a**^	
		**β**	***P***	**β**	***P***
*Individual cognitions*					
	Attitude	***−0.10***	<.001*	***0.18***	<.001*
	Perception whether vegetables are expensive	−0.04	.12	**−0.05**	.02*
	Subjective norm	−0.003	.90	−0.02	.40
	Self-efficacy	−0.02	.56	**0.20**	<.001*
	Intention	−0.04	.18	**0.23**	<.001*
*Self-regulation concepts*					
	General self-regulation	**−0.14**	<.001*	0.04	.08
	Action planning	**0.08**	.004*	0.02	.48
	Coping planning	0.03	.33	−0.06	.09
*Environmental-level factors*					
	Perception of availability in supermarket	0.001	.96	**−0.05**	.02*
	Availability of vegetables at home	***−0.10***	<.001*	***0.16***	<.001*

*Significant association (*P* < .05).

^a^Adjusted for age, sex, place of residence and ethnicity; ^b^Adjusted for education.

**Bold**: significant association for path a or path b; ***Bold and italic:*** significant association for both paths.

### Total effect, direct effect and percentage mediated effect

The multiple linear regression analysis showed that there was a significant, negative, association between education and vegetable consumption (path c; β = −0.15, *P* < .001), indicating that low/moderate-educated individuals have a lower vegetable consumption than high-educated individuals. When all potential mediators were included in the multiple linear regression model, the association between education and vegetable consumption remained significant (path c’; β = −0.10, *P* < .001), indicating partial mediation by attitude and availability of vegetables at home. The same result was found when only attitude and availability of vegetables at home, and not the other potential mediators, were included in the model (β = −0.08, *P* = .001).

Attitude and availability of vegetables at home explained 24.46% of the association between education and vegetable consumption (12.84% was mediated by attitude and 11.62% by availability of vegetables at home). Calculating the percentage mediated effect for the separate attitude items showed that the health, importance and taste beliefs mediated 1.08%, 4.21% and 7.35% respectively.

The bootstrapping verification procedure produced similar results.

## Discussion

This study explored whether individual cognitions, self-regulation and physical environmental factors can explain the difference in vegetable consumption between low/moderate- and high-educated adults in the Netherlands. Attitude towards consuming 200 grams of vegetables a day and availability of vegetables at home were found to partially mediate this relation.

The finding that attitude mediated the association between education and vegetable consumption is in line with the findings of a previous study on fruit consumption among adolescents [19]. Although both educational groups had a positive attitude towards vegetable consumption, the attitude of low/moderate-educated individuals was (slightly) less positive. This small difference apparently contributes to an educational difference in vegetable consumption. No other studies that assess a mediating effect of attitude were found, but this finding indicates that attitude is important in the association between education and vegetable consumption. Since beliefs about taste had the largest percentage mediated effect of the three attitude items, it may be most beneficial to target taste perceptions. However, more studies are needed to confirm this finding.

Availability of vegetables at home also mediated the association between education and vegetable consumption, which is in line with a previous study among adolescents [25]. Having vegetables at home less often was associated with a lower vegetable consumption and, although availability of vegetables at home was high for both educational groups, the availability at home was (slightly) lower for low/moderate-educated individuals. Again, this apparently small difference can result in an educational difference in vegetable consumption. Although the physical environment is often suggested to be important for dietary behavior, this study suggests that, with respect to vegetable consumption, it can also be the physical home environment that is of bigger influence than the broader physical neighborhood environment.

Perceived availability of vegetables in the supermarket was not a mediator of the difference in vegetable consumption, since this perception did not differ between the two educational groups. An explanation for this may be that in the Netherlands differences in (objective) availability of vegetables in supermarkets between high and low socio-economic neighborhoods are not that prominent, as is also suggested in another Dutch study that did also not find educational differences in this perception [23]. Another explanation may be that both low/moderate- and high-educated individuals have chosen to buy their vegetables in a specific supermarket because this supermarket has a sufficient amount of vegetables available. This could decrease educational differences in this perception.

Contrary to our expectations, no evidence for a mediating effect was found for self-efficacy, subjective norm, intention and the perception of vegetables as being expensive, mainly because no educational differences were found. This is in contrast with other studies that did find educational differences in variables such as relative importance of price [26], perceived costs of vegetables [9,23], self-efficacy [17] and perceived barriers related to vegetable consumption [18]. Participants in our study were recruited to participate in a study evaluating a nutrition education intervention. This could have led to including a selective group of individuals who were motivated and willing to increase vegetable consumption, which may explain not finding educational differences in these variables. This suggestion is supported by the finding that vegetable consumption was higher and educational differences herein were smaller than in the general Dutch population [2].

For general self-regulation, educational differences were found, but the association with vegetable consumption was only borderline significant (*P* = 0.08). This could be due to assessing general instead of vegetable-specific self-regulation skills. A study of Anderson et al. [20] assessed vegetable-specific self-regulatory behaviors and found that enactment of self-regulatory behaviors, such as regulating vegetable consumption, had a positive effect on vegetable consumption. However, the self-regulation concepts action- and coping planning were measured vegetable-specific in our study but were also not associated with vegetable consumption. This latter finding may be due to the measurement of action- and coping planning. Individuals only had to report whether they have plans, without a prior manipulation or instruction of making plans, which is in contrast to other studies that showed associations of action- and coping planning with vegetable consumption (e.g. [21]). Consequently, we have measured plans that individuals have formed ‘spontaneously’. The quality of such plans may not be optimal, resulting in a lack of association with vegetable consumption. Another explanation may be that the plans were formed for increasing vegetable consumption in the future, which makes it less likely that these plans are associated with current behavior. Action planning was, however, associated with education. Although the difference was only small, low/moderate-educated individuals reported to have more often plans on when, how much and which vegetables to eat more. This may also be explained by the assessment of the concept. We measured planning regarding going to eat more vegetables. Since low/moderate-educated individuals consumed less vegetables, they may also have had more opportunities to improve vegetable consumption than high-educated individuals and may therefore have been more likely to have plans for eating more vegetables.

The educational difference in daily vegetable consumption in our study was, although significant, only 16.8 grams. This is smaller than the educational difference in the general Dutch population [2]. One explanation for this may be that our study sample was highly motivated, which is supported by the high intention to consume at least 200 grams of vegetables a day. Although a difference of 16.8 grams per day seems to be small, it comes to a difference in weekly consumption of 117.6 grams, which is still relevant.

Attitude and availability of vegetables at home did not completely mediate the association between education and vegetable consumption. This indicates that the effect is mainly direct or that other factors, such as storage [37], social environment [38] and taste preferences [39] may also be mediators. More research on other potentially important factors is needed, in order to get insight in other factors that contribute to educational differences in vegetable consumption.

### Limitations

Several limitations should be taken into account when interpreting the results of this study. Firstly, because of the cross-sectional design of the study no conclusions on causality or directions of the associations can be drawn. Secondly, although a validated questionnaire was used to measure vegetable consumption [28,29], measurements were self-reported. This may be less accurate than using more objective instruments, such as biomarkers, and may have resulted in, for example, response bias or over-reporting. Using objective instruments was, however, not feasible in this study because of the large number of participants. In addition, the questionnaires were validated for hard-copy use only, while we used online versions. Thirdly, most potential mediators were measured with a small number of items and not all variables could be measured with validated or existing questionnaires. Therefore, new questions were developed for this study, for which validity is unknown. Also, the validity and reliability of measures among low-educated individuals is unknown. This may be especially the case for action- and coping planning, since these are complex concepts that may be hard to interpret. Another limitation could be that we may have recruited a selective sample, since only a very small proportion of the study population had a non-Western background and the consumption levels of both educational groups are high compared to the general Dutch population [2]. In addition, the educational groups differed in age and percentage male. By adjusting the analyses for, amongst others, these variables, the results are probably still reliable. Lastly, since only 19.3% of the study sample were low-educated and differences between low and moderate educational groups are reported to be small [2], low- and moderate-educated individuals were combined into one group. This may have resulted in smaller differences between educational groups. Therefore, future research that focuses on educational differences should include more low-educated individuals.

### Conclusion and practical implications

Attitude and availability of vegetables at home explained about 25% of the educational difference in vegetable consumption in this sample of Dutch adults. Low/moderate-educated individuals had a less positive attitude towards consuming 200 grams of vegetables a day and had less often vegetables available at home, which both were related to a lower vegetable consumption. Although longitudinal and replicated studies are needed to further test the findings of this study, these results indicate that interventions aimed at decreasing educational differences in vegetable consumption should focus on promoting a positive attitude and increasing the availability of vegetables at home among low/moderate-educated individuals.

## Abbreviations

CVD: Cardiovascular diseases
TPB: Theory of Planned Behavior

## References

[1] van Bakel AM (2013). Voeding Samengevat (Nutrition Summarized). Volksgezondheid Toekomst Verkenning, Nationaal Kompas Volksgezondheid.

[2] van Rossum CTM, Fransen HP, Verkaik-Kloosterman J, Buurma-Rethans EJM, Ocké MC (2011). Dutch National Food Consumption Survey 2007–2010: Diet of children and adults aged 7 to 69 years.

[3] van Kranen HJ, van Raaij JMA, van Bakel AM (2013). Wat is de relatie tussen voeding en gezondheid? (What is the relation between nutrition and health?). Volksgezondheid Toekomst Verkenning, Nationaal Kompas Volksgezondheid.

[4] Centers for Disease Control and Prevention (2013). State Indicator Report on Fruits and Vegetables, 2013.

[5] Lock K, Pomerleau J, Causer L, Altmann DR, McKee M (2005). The global burden of disease attributable to low consumption of fruit and vegetables: implications for the global strategy on diet. Bull World Health Organ.

[6] De Irala-Estévez J, Groth M, Johansson L, Oltersdorf U, Prättälä R, Martínez-González MA (2000). A systematic review of socio-economic differences in food habits in Europe: consumption of fruit and vegetables. Eur J Clin Nutr.

[7] van Rossum CTM, Geurts M (2013). Voeding: Zijn er Verschillen Naar Sociaaleconomische Status? (Nutrition: Are There Differences to Socio-Economic Status?). Volksgezondheid Toekomst Verkenning, Nationaal Kompas Volksgezondheid.

[8] Ball K, Crawford D, Mishra G (2006). Socio-economic inequalities in women’s fruit and vegetable intakes: a multilevel study of individual, social and environmental mediators. Public Health Nutr.

[9] Inglis V, Ball K, Crawford D (2008). Socioeconomic variations in women’s diets: what is the role of perceptions of the local food environment?. J Epidemiol Community Health.

[10] Ajzen I (1991). The theory of planned behavior. Organ behav hum dec.

[11] Bandura A (1986). Social Foundations of Thought and Action: A Social Cognitive Theory.

[12] Maes S, Karoly P (2005). Self-regulation assessment and intervention in physical health and illness: a review. Appl Psychol, Int Rev.

[13] Kremers SPJ, de Bruijn G-J, Visscher TLS, van Mechelen W, de Vries NK, Brug J (2006). Environmental influences on energy balance-related behaviors: a dual-process view. Int J Behav Nutr Phys Act.

[14] Sjoberg S, Kim K, Reicks M (2004). Applying the Theory of Planned Behavior to Fruit and Vegetable Consumption by Older Adults. J Nutr Elder.

[15] Kvaavik E, Lien N, Tell GS, Klepp K-I (2005). Psychosocial predictors of eating habits among adults in their mid-30s: The Oslo Youth Study follow-up 1991–1999. Int J Behav Nutr Phys Act.

[16] Shaikh AR, Yaroch AL, Nebeling L, Yeh M-C, Resnicow K (2008). Psychosocial Predictors of Fruit and Vegetable Consumption in Adults: A Review of the Literature. Am J Prev Med.

[17] Sherer M, Maddux JE, Mercandante B, Prentice-Dunn S, Jacobs B, Rogers RW (1982). The self-efficacy scale: construction and validation. Psychol Rep.

[18] Dittus KL, Hillers VN, Beerman KA (1995). Benefits and Barriers to Fruit and Vegetable Intake: Relationship between Attitudes and Consumption. J Nutr Educ.

[19] Ball K, MacFarlane A, Crawford D, Savige G, Andrianopoulos N, Worsley A (2009). Can social cognitive theory constructs explain socio-economic variations in adolescent eating behaviours? A mediation analysis. Health Educ Res.

[20] Anderson ES, Winett RA, Wojcik JR (2007). Self-Regulation, Self-Efficacy, Outcome Expectations, and Social Support: Social Cognitive Theory and Nutrition Behavior. Ann Behav Med.

[21] Wiedemann AU, Lippke S, Reuter T, Ziegelmann JP, Schwarzer R (2011). How planning facilitates behaviour change: Additive and interactive effects of a randomized controlled trial. Eur J Soc Psychol.

[22] van Osch L, Lechner L, Reubsaet A, de Vries H (2010). From theory to practice: An explorative study into the instrumentality and specificity of implementation intentions. Psychol Health.

[23] Giskes K, van Lenthe FJ, Kamphuis CBM, Huisman M, Brug J, Mackenbach JP (2009). Household and food shopping environments: do they play a role in socioeconomic inequalities in fruit and vegetable consumption? A multilevel study among Dutch adults. J Epidemiol Community Health.

[24] Rasmussen M, Krølner R, Klepp K-I, Lytle L, Brug J, Bere E, Due P (2006). Determinants of fruit and vegetable consumption among children and adolescents: a review of the literature. Part I: quantitative studies. Int J Behav Nutr Phys Act.

[25] Bere E, van Lenthe F, Klepp K-I, Brug J (2008). Why do parents’ education level and income affect the amount of fruits and vegetables adolescents eat?. Eur J Public Health.

[26] Konttinen H, Sarlio-Lähteenkorva S, Silventoinen K, Männistö S, Haukkala A (2012). Socio-economic disparities in the consumption of vegetables, fruit and energy-dense foods: the role of motive priorities. Public Health Nutr.

[27] Springvloet L, Lechner L, Oenema A (2014). Planned development and evaluation protocol of two versions of a web-based computer-tailored nutrition education intervention aimed at adults, including cognitive and environmental feedback. BMC Public Health.

[28] Bogers RP, van Assema P, Kester ADM, Westerterp KR, Dagnelie PC (2004). Reproducibility, Validity, and Responsiveness to Change of a Short Questionnaire for Measuring Fruit and Vegetable Intake. Am J Epidemiol.

[29] van Assema P, Brug J, Ronda G, Steenhuis I, Oenema A (2002). A short dutch questionnaire to measure fruit and vegetable intake: relative validity among adults and adolescents. Nutr Health.

[30] Verweij A (2008). Onderwijsdeelname: Indeling opleidingsniveau (Education participation: Classification level of education). Volksgezondheid Toekomst Verkenning, Nationaal Kompas Volksgezondheid.

[31] Carey KB, Neal DJ, Collins SE (2004). A psychometric analysis of the self-regulation questionnaire. Addict Behav.

[32] Sniehotta FF, Schwarzer R, Scholz U, Schüz B (2005). Action planning and coping planning for long-term lifestyle change: Theory and assessment. Eur J Soc Psychol.

[33] Keij I (2000). Standaarddefinitie allochtonen (Standard definition immigrants). Hoe doet het CBS dat nou? (How does Statistics Netherlands do this?).

[34] MacKinnon DP, Lockwood CM, Hoffman JM, West SG, Sheets V (2002). A Comparison of Methods to Test Mediation and Other Intervening Variable Effects. Psychol Methods.

[35] Fritz MS, MacKinnon DP (2007). Required Sample Size to Detect the Mediated Effect. Psychol Sci.

[36] Field A, Field A (2009). Exploring assumptions. Discovering statistics using SPSS.

[37] Giskes K, Turrell G, Patterson C, Newman B (2002). Socio-economic differences in fruit and vegetable consumption among Australian adolescents and adults. Public Health Nutr.

[38] Sorensen G, Stoddard AM, Dubowitz T, Barbeau EM, Bigby J, Emmons KM, Berkman LF, Peterson KE (2007). The Influence of Social Context on Changes in Fruit and Vegetable Consumption: Results of the Healthy Directions Studies. Am J Public Health.

[39] Murcott A (2002). Nutrition and inequalities. A note on sociological approaches. Eur J Public Health.

